# Impact of background music listening on anxiety in cancer patients undergoing initial radiation therapy: a randomized clinical trial

**DOI:** 10.1186/s13014-024-02460-3

**Published:** 2024-06-11

**Authors:** Huei-Fan Yang, Wen-Wei Chang, Ying-Hsiang Chou, Jing-Yang Huang, Yu-Shiun Liao, Ting-En Liao, Hsien-Chun Tseng, Shih-Tsung Chang, Hsin Lin Chen, Ya-Fang Ke, Pei-Fang Tsai, Hsiu-Man Chan, Bo-Jiun Chang, Yi-Ting Hwang, Hsueh-Ya Tsai, Yueh-Chun Lee

**Affiliations:** 1 Department of Radiation Oncology, Chung Shan Medical University Hospital, No.110, Sec.1, Jianguo N. Rd, Taichung, 40201 Taiwan; 2 Department of Nursing, Chung Shan Medical University Hospital, Taichung, 40201 Taiwan; 3 Department of Biomedical Sciences, Chung Shan Medical University, Taichung, 40201 Taiwan; 4 Department of Medical Imaging and Radiological Sciences, Chung Shan Medical University, Taichung, 40201 Taiwan; 5 Institute of Medicine, Chung Shan Medical University, Taichung, 40201 Taiwan; 6 Department of Medical Research, Chung Shan Medical University Hospital, Taichung, 40201 Taiwan; 7 Taichung Municipal Chu-Jen Junior High School, Taichung, 403002 Taiwan; 8 School of Medicine, Chung Shan Medical University, Taichung, 40201 Taiwan

**Keywords:** Radiation therapy, Music listening intervention, Anxiety symptoms, Cancer patients

## Abstract

**Background:**

Patients undergoing radiation therapy (RT) often experience anxiety, which may jeopardize the treatment success. The efficacy of music interventions in reducing anxiety remains contentious. This randomized trial aimed to evaluate the impact of music listening on anxiety symptoms in patients undergoing initial RT.

**Methods:**

First-time RT patients were randomly allocated to experimental and control groups. The Brief Symptom Rating Scale (BSRS-5), Distress Thermometer (DT), and Beck Anxiety Inventory (BAI-C) were administered pre- and post-RT. Changes in physiological anxiety symptoms were monitored over 10 consecutive days starting from the first day of RT. The experimental group received music during RT; the control group did not. The generalized linear mixed model was used to estimate the pre–post difference in the BSRS-5, DT, and BAI-C scores between the music intervention and control group.

**Results:**

This study included 50 patients each in the experimental and control groups. BSRS-5 and DT scores were significantly reduced in the experimental group post-RT (*p* = 0.0114 and *p* = 0.0023, respectively). When music listening was discontinued, these scores rebounded. While the posttest BAI-C score was significantly lower in the experimental group (*p* < 0.0001), the pre–post difference between the two groups was not significant (*p* = 0.0619). On cessation of music listening, the BAI-C score also rebounded.

**Conclusions:**

For cancer patients undergoing initial RT, music listening intervention significantly reduced anxiety symptoms measured using the BSRS-5, DT, and BAI-C scores after two weeks. Our results demonstrate the effectiveness of music listening intervention in reducing anxiety symptoms, thereby potentially improving the quality of life of cancer patients undergoing RT.

## Background

Malignant tumors are the leading cause of death in Taiwan, and radiation therapy (RT), which employs ionizing radiation, is a crucial component of the multimodal approach to cancer treatment [1]. Despite its efficacy in targeting various malignant tumors and potential applications in treating certain benign diseases and disorders, RT often induces anxiety in patients, especially in those experiencing it for the first time. This anxiety, sparked by anticipatory worries about the potential side effects, unfamiliarity with the treatment equipment, long waiting times, and feelings of loneliness during treatment, may result in treatment interruption in approximately 25% of patients [2–5]. In response to this challenge, music interventions have emerged as promising noninvasive techniques for alleviating stress and pain, relaxing muscles, and enhancing patients’ emotional management capabilities [6]. Music interventions, which encompass activities like listening to familiar music, singing, creating, and improvising, have demonstrated positive impact on emotional regulation and anxiety reduction. Integrating psychosocial interventions such as music therapy into comprehensive cancer care addresses not only the physical symptoms but also the psychological and emotional well-being of patients. These interventions have been shown to enhance patients’ overall well-being, quality of life, and coping abilities [7–10]. A growing body of research highlights high levels of anxiety among women undergoing RT for cancer [11, 12]. However, both national and international literature on the anxiety experienced by patients undergoing RT remains limited. Furthermore, the introduction of music listening interventions during RT for oncology showed anxiety reduction only during a single session in the simulation phase [11], while a single session of music intervention during the first radiation treatment did not show anxiety reduction [11]. Music intervention utilizes specific sound waves to create a peaceful state for patients [12, 13]. It reduces anxiety by influencing the stress hormone release. Relaxing music positively impacts the autonomic nervous system, thus stabilizing the breathing, heart rate, blood pressure, and pulse. It also affects neurotransmitters, hormones, and the endocrine system, leading to pain relief, stress reduction, improved mood, relaxation, enhanced spirituality, and better communication [14]. The effect of music on various physiological functions requires further research. Music transmission occurs through the limbic and hypothalamic systems, contributing to the psychological effects [16–19]. Studies have shown that music interventions can alleviate pain and anxiety in burn patients [20]. Music intervention may also benefit cancer patients, improving the anxiety levels, heart rate, respiratory rate, and blood pressure [21].

Based on previous research demonstrating the efficacy of music interventions in reducing anxiety levels [13–24], we hypothesize here that background music listening during the RT process may have an anxiolytic effect in patients undergoing RT for the first time. The aim of this study was to investigate the potential of background music listening to reduce anxiety and nervous behavior in Taiwanese patients undergoing RT using a prospective randomized trial.

## Methods

### Study design

Participants were randomly assigned to two groups using the random assignment software provided by IClinical developed by the Office Of data Science, Taipei Medical University (http://biostat.tmu.edu.tw/iclinical/).

### Research site and participants

Participants were recruited from the Department of Radiation Oncology at Chung Shan Medical University Hospital (Taichung City, Taiwan). The inclusion criteria were as follows: (a) age 18 years or older, (b) patients undergoing RT for cancer for the first time, (c) patients with clear consciousness and normal hearing, and (d) patients willing to respond to the questionnaire after the research process and purpose were explained to them. The exclusion criteria included: (a) patients who had previously undergone radiation treatment, (b) patients with diminished mental capacity or inability to satisfactorily participate in this study, and (c) patients with moderate to severe hearing impairment. A total of 100 participants were enrolled and randomly assigned to either the music group or nonmusic group, with 50 individuals in each group, and there were no withdrawals or involuntary enrolments during the study. We ensured an equal distribution of men and women, without considering the type of cancer. To minimize the confounding factors and prevent shared learning experiences or communication between the two groups, patients in the experimental and control groups were scheduled for treatment at different times throughout the year (from January 1, 2022 to December 31, 2022). Of the patients enrolled, 21 in the control group and 17 in the experimental group received neoadjuvant chemotherapy; 14 in the control group and 15 in the experimental group received concurrent chemoradiotherapy; 15 in the control group and 18 in the experimental group received radiotherapy alone. Anxiety assessment questionnaires were administered before the treatment began and after 2 weeks of treatment. Physiological parameters, including heart rate, systolic and diastolic blood pressure, and blood oxygen levels, were monitored before and after 2 weeks of treatment. In the background music listening group, patients received music intervention during the treatment for a consecutive period of 10 days. They listened to music for approximately 10–15 min. To comply with the infection control protocols, music was delivered through broadcast speakers instead of headphones. This period was then followed by a 10-day period without music during treatment in order to compare the differences in the same individuals. In contrast, the control group did not receive music intervention throughout their entire RT period. We observed the music intervention group for 2 weeks to ascertain any differences.

### Background music listening during RT

In our study, patients completed the necessary questionnaires before receiving an individual music therapy intervention for approximately 10–15 min per session. The music used in the intervention was selected from an MP3 music file database that included over 360,000 downloads of relaxing piano and violin music and was played using a Bluetooth speaker. During the treatment, the music group listened to the selected music through speaker playback. The intervention was conducted consecutively for a period of 10 days.

### Outcome assessment

The physiological indicators, including heart rate, blood pressure, and blood oxygen, were measured by a PHILIPS M1205A noninvasive physiological monitor. The anxiety status of the enrolled patients was assessed by questionnaires, which included the following: (1) Distress Thermometer (DT): Screening tool for psychological distress in cancer patients recommended by the NCCN [25]; (2) Brief Symptom Rating Scale (BSRS-5) or “Mood Thermometer”: 5-point Likert scale for assessing symptoms of emotional distress over one week. The total score ranges from 0 to 20 [26, 27]; (3) Beck Anxiety Inventory (BAI-C): A 21-item self-reported questionnaire assessing anxiety symptoms on a 4-point scale, which has shown excellent internal consistency and test-retest reliability; (4) Symptom Distress Thermometer (SDT): A 10-point scale by the NCCN to evaluate distress levels, with scores ≥ 4 indicating clinically significant distress. These assessment tools provided insights into the anxiety and distress levels among the participants during the study [28, 29].

### Statistical analyses

Primary analysis: Independent t-test was used for comparing the percentage reduction in the mean BAI-C scores between the music and nonmusic groups. It aimed to detect a minimum 20% reduction in the mean BAI score in the music group compared to no change in the nonmusic group [30]. Generalized linear mixed models were employed to analyze the within-between interaction [31].

Secondary analysis: We assessed the normality of BAI-C, DT, and BSRS-5 scores using the Shapiro-Wilk and Kolmogorov-Smirnov tests. The normality assumption for BAI-C, DT, and BSRS-5 scores was violated. Therefore, the Mann-Whitney U test was utilized to compare the difference of BAI-C, DT, and BSRS-5 scores between the music and non-music groups at baseline and post-radiotherapy (RT). A Wilcoxon’s signed-rank test was conducted to evaluate the differences between baseline and post-RT scores, treating these as repeated measures.

By employing these statistical approaches, the effects of music therapy on anxiety (BAI-C) and distress (SDT) levels among the participants were examined comprehensively [30, 31]. The BAI-C showed moderate correlation with other measures and was able to discriminate between groups with different diagnoses. It is considered to be a reliable psychometric inventory suitable for use in inpatient and outpatient settings [32–35]. The range of BAI-C scores is 0–63. The total score is interpreted as follows: 0–7 is considered minimal, 8–15 mild, 16–25 moderate and 26–63 severe, following a previous report by Fydrich et al. [36]. The cut-off value for the BAI-C was set at 8, as 8 (16%) patients in the music group and 18 (36%) in the control group had a pre-BAI-C score of ≥ 8.

This study conducted data analysis using SAS 9.4 software (SAS Institute Inc. SAS 9.4. 2014, SAS Institute Inc.). All tests were two-tailed, and a p-value of < 0.05 was considered statistically significant.

## Results

### Baseline characteristics of the study participants

This study enrolled 50 patients each in the music intervention and control groups. Table 1 presents the baseline characteristics of the participants, including demographics (sex, age, marital status, living status, and education level), lifestyle habits (smoking, alcohol drinking, and betel nut chewing), frequency of listening to music per week, favorite music genre, body mass index, co-morbidity (diabetes mellitus, hypertension, cardiovascular disease), use of sedative-hypnotic drugs, type of cancer, cancer stage, surgical treatment for cancer, mask/mold, radiation dose, frequency of radiation, and whether the treatment was curative or palliative. There were no significant differences in these baseline characteristics between the music intervention and control groups.

**Table 1:** Baseline characteristics among the music group and non-music group

	Music group	Non-music group	*p* value
N	50	50	
Female	25 (50.0%)	27 (54.0%)	0.6889
Age	59.9 ± 9.6	60.3 ± 11.3	0.8643
Marital status			0.8415
Single	6 (12.0%)	6 (12.0%)	
Married	42 (84.0%)	43 (86.0%)	
Divorced	2 (4.0%)	1 (2.0%)	
Living status			0.4870
Live with spouse or children	44 (88.0%)	47 (94.0%)	
Living alone or other type	6 (12.0%)	3 (6.0%)	
Education			0.1574
≦ 12 years	25 (50.0%)	18 (36.0%)	
> 12 years	25 (50.0%)	32 (64.0%)	
Life style			
Smoking	22 (44.0%)	22 (44.0%)	1.0000
Alcohol drinking	14 (28.0%)	14 (28.0%)	1.0000
Betel nut chewing	19 (38.0%)	11 (22.0%)	0.0809
Listen to music frequency			0.0639
Every day	16 (32.0%)	14 (28.0%)	
A few days a week	19 (38.0%)	10 (20.0%)	
Rarely listened to	12 (24.0%)	16 (32.0%)	
not	3 (6.0%)	10 (20.0%)	
Prefer music genres			
Musical instruments light music	19 (38.0%)	17 (34.0%)	0.6769
Taiwanese old song	29 (58.0%)	30 (60.0%)	0.8389
popular music	12 (24.0%)	12 (24.0%)	1.0000
Rock jazz	0 (0.0%)	2 (4.0%)	0.1531
BMI	22.6 ± 4.4	24.1 ± 5.5	0.1637
Co-morbidity			
Diabetes mellitus	16 (32.0%)	12 (24.0%)	0.3730
Hypertension	12 (24.0%)	14 (28.0%)	0.6484
Cardiovascular disease	2 (4.0%)	7 (14.0%)	0.0806
Sedative-hypnotics drug	10 (20.0%)	11 (22.0%)	0.8061
Cancer type			0.9118
Head and neck cancer	14 (28.0%)	15 (30.0%)	
Breast cancer	14 (28.0%)	14 (28.0%)	
Lung cancer	10 (20.0%)	10 (20.0%)	
Colorectal cancer	4 (8.0%)	3 (6.0%)	
Gynecological cancer	7 (14.0%)	5 (10.0%)	
Other	1 (2.0%)	3 (6.0%)	
Cancer stage			0.4938
I	10 (20.0%)	8 (16.0%)	
II	11 (22.0%)	18 (36.0%)	
III	14 (28.0%)	12 (24.0%)	
IV	15 (30.0%)	12 (24.0%)	
Surgery treatment for cancer	43 (86.0%)	40 (80.0%)	0.4245
Mask/Mold			0.2797
Mask	18 (36.0%)	13 (26.0%)	
Mold	32 (64.0%)	37 (74.0%)	
Radiation dose	58.3 ± 6.2	57.6 ± 6.5	0.5935
Number of radiations	27.6 ± 5.4	27.6 ± 5.2	0.9851
Curative/Palliative			0.3074
Curative	47 (94.0%)	49 (98.0%)	
Palliative	3 (6.0%)	1 (2.0%)	

### Outcome of the beck anxiety inventory

**Fig. 1:**
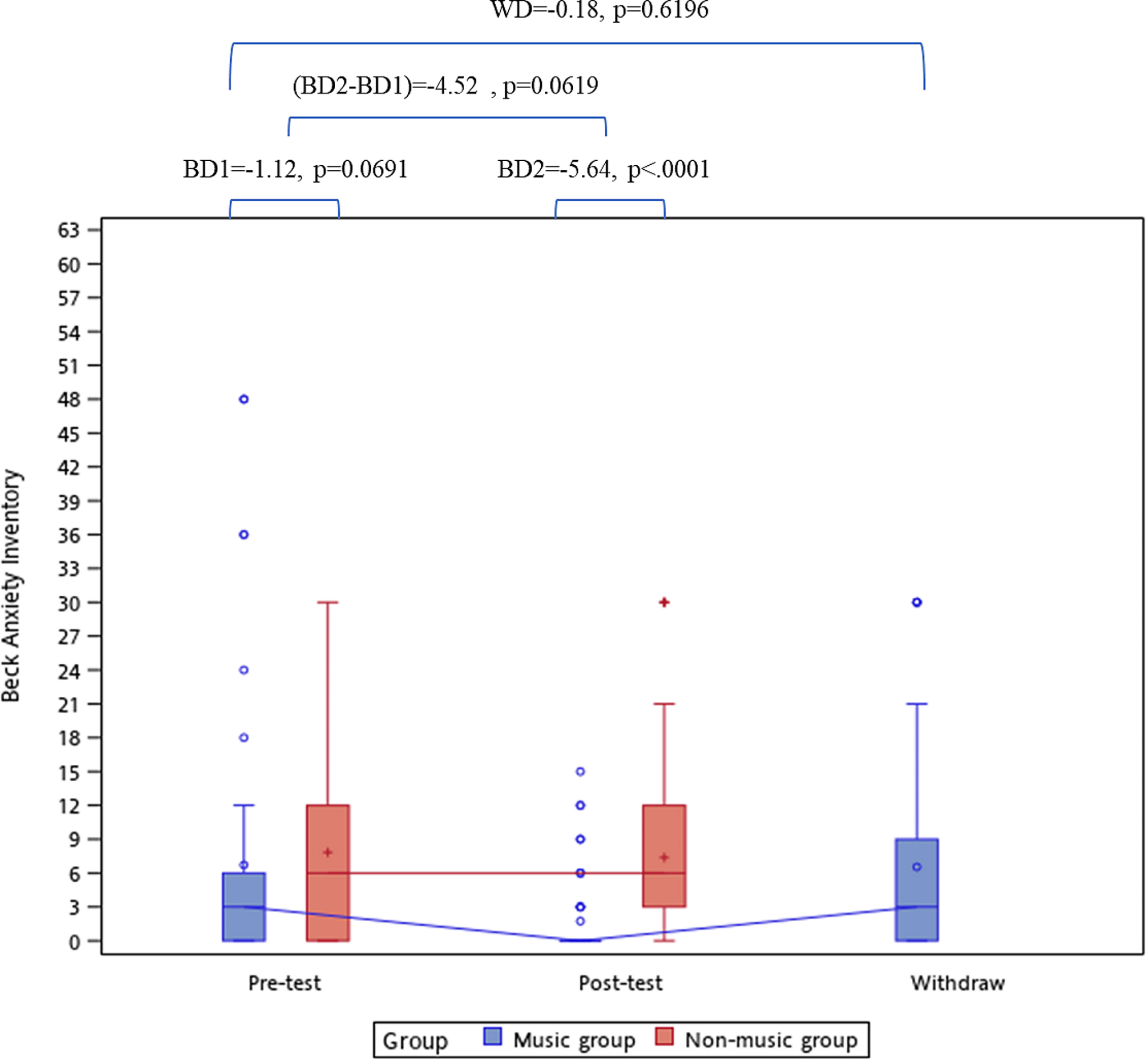
The between-group and within-group difference of BAI-C. BD1, the difference of BAI-C between music group and non-music group during pre-test, and compared by Mann-Whitney U test. BD2, the difference of BAI-C between music group and non-music group during post-test, and compared by Mann-Whitney U test (BD2-BD1), the pre-post difference of BAI-C between music group and non-music group, and estimated by generalized linear mixed model. WD, the difference of BAI-C in music group after withdraw the background music listening compared with pre-test, and estimated by Wilcoxon’s signed rank test

The pre-test BAI-C scores were 6.7 ± 11.7 and 7.8 ± 8.5 in the music and control groups, respectively, with a group difference of − 1.12 (*p* = 0.5878). The posttest BAI-C scores were 1.7 ± 3.7 and 7.4 ± 8.0 in the music and control groups, respectively, with a group difference of − 5.64 (*p* < 0.0001, Table 2). Figure 1 shows that the difference between the pre- and posttest scores was − 4.52 (*p* = 0.0619) between the groups. When the music group stopped receiving the music listening, the BAI-C rebounded to 6.5 ± 8.7, which was not significantly different from the pre-test BAI-C score in the music group (*p* = 0.9300). Table 3 displays the proportion of post-BAI-C scores ≥ 8 in the music and control groups, stratified by the level of pre-BAI-C. In patients with pre-BAI-C ≥ 8, the prevalence of post-BAI-C ≥ 8 was 62.5% in the music group and 94.4% in the control group (*p* = 0.0372). In patients with pre-BAI-C < 8, the prevalence of post-BAI-C ≥ 8 was 0.0% in the music group and 3.1% in the control group (*p* = 0.4324). The Cochran Q was 32.04 (*p* < 0.0001), indicating a significant difference in the marginal prevalence of post-BAI-C ≥ 8 between the groups. In the patients who had pre-BAI-C ≥ 8, the marginal prevalence (94.4–62.5% = 36.9%) was significantly different from those who had pre-BAI-C < 8 (3.1–0.0% = 3.1%).

**Table 2:** The distribution of Beck Depression Inventory, Symptom Distress Thermometer, and Brief Symptom Rating Scale-5 among music group and non-music group

	Mean ± SD				
	Music group	Non-music group	*p* value^‡^	*p* value^†^	*p* value^#^
N	50	50			
Beck anxiety Inventory					
Pre-test	6.7 ± 11.7	7.8 ± 8.5	0.0691	-	-
Post-test	1.7 ± 3.7	7.4 ± 8.0	< 0.0001	< 0.0001	0.5794
Withdraw-music	6.5 ± 8.7	-	-	0.6196	-
Symptom Distress Thermometer					
Pre-test	4.3 ± 1.5	3.4 ± 1.7	0.0076	-	-
Post-test	2.7 ± 1.2	3.5 ± 1.3	0.0023	< 0.0001	0.5086
Withdraw-music	3.7 ± 1.4	-	-	0.1009	-
Brief Symptom Rating Scale-5					
Pre-test	3.4 ± 2.3	2.6 ± 2.3	0.0453	-	-
Post-test	1.6 ± 1.4	2.7 ± 2.2	0.0114	< 0.0001	0.6931
Withdraw-music	3.7 ± 3.0	-	-	1.0000	-

^‡^ The p value indicated the significance of mean difference between music group and non-music group by Mann-Whitney U test

^†^ The p value indicated the significance of mean difference compared with pre-test value within music group by Wilcoxon’s signed rank test

^#^The p value indicated the significance of mean difference compared with pre-test value within non-music group by Wilcoxon’s signed rank test

**Table 3:** The prevalence of post-BAI-C ≥ 8 between music group and non-music group, stratified by the level of pre-BAI-C

Cochran Q = 32.04, *p* < 0.0001	Pre-BAI-C ≥ 8			Pre-BAI-C < 8		
	Music group	Non-music group	p value^‡^	Music group	Non-music group	p value^‡^
N	8	18		42	32	
n(% ) of post-BAI-C ≥ 8	5(62.5%)	17(94.4%)	0.0372	0(0.0%)	1(3.1%)	0.4324

^‡^ The p value indicated the significance of heterogeneity of high post-BAI-C prevalence between music group and non-music group by chi-square test and stratified by the level of pre-BAI-C. The Cochran-Mantel-Haenszel Statistics was performed, the Cochran Q was 32.04 and *p* < 0.0001, the result indicated the marginal prevalence (94.4%-62.5%=36.9%) of post-BAI-C ≥ 8 between groups in patients had pre-BAI-C ≥ 8 was significantly different from (3.1%-0.0%=3.1%) in the patients had pre-BAI-C < 8

### Outcome of the symptom distress thermometer

**Fig. 2:**
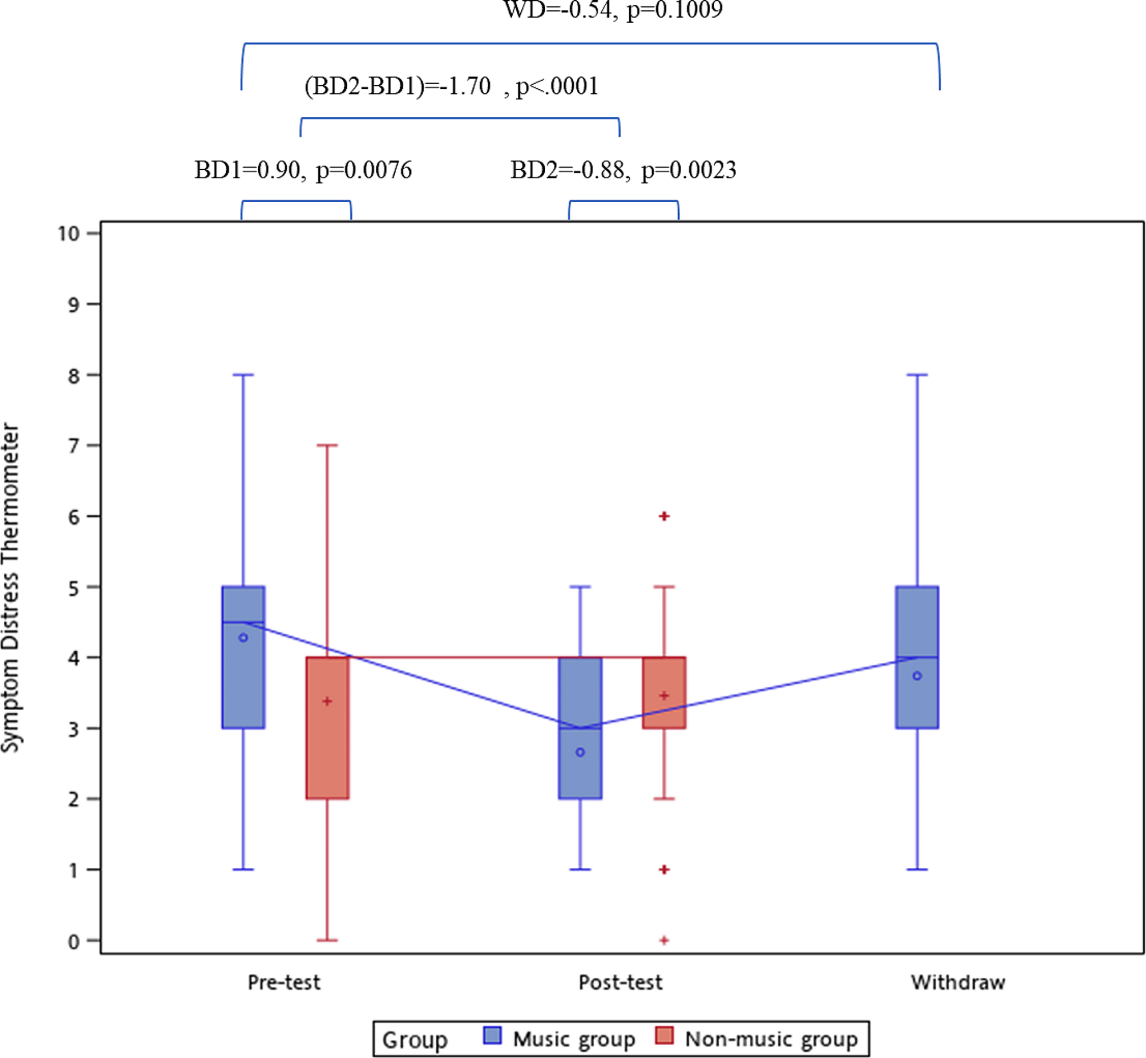
The between-group and within-group difference of DT. BD1, the difference of DT between music group and non-music group during pre-test, and compared by Mann-Whitney U test. BD2, the difference of DT between music group and non-music group during post-test, and compared by Mann-Whitney U test. (BD2-BD1), the pre-post difference of DT between music group and non-music group, and estimated by generalized linear mixed model. WD, the difference of DT in music group after withdraw the background music listening compared with pre-test, and estimated by Wilcoxon’s signed rank test

The pre-test SDT scores were 4.3 ± 1.5 in the music group and 3.4 ± 1.7 in the control group, resulting in a group difference of + 0.90 (*p* = 0.0051). The posttest SDT scores were 2.7 ± 1.2 in the music group and 3.5 ± 1.3 in the control group, resulting in a group difference of − 0.88 (*p* = 0.0021, Table 2). Figure 2 shows that the pre–post SDT difference was − 1.70 (*p* < 0.0001) between the groups. After discontinuing the music listening intervention, the SDT rebounded to 3.7 ± 1.4, which was not significantly different from the pre-test SDT in the music group (*p* = 0.0662). Table 4 shows the prevalence of post-SDT scores ≥ 4 in the music and control groups, stratified by the level of pre-SDT. In patients with pre-SDT ≥ 4, the prevalence of post-SDT ≥ 4 was 42.4% in the music group and 78.6% in the control group (*p* = 0.0042). In patients with pre-SDT < 4, the prevalence of post-SDT ≥ 4 was 0.0% in the music group and 22.7% in the control group (*p* = 0.0565). The Cochran Q was 9.87 (*p* = 0.0072), indicating a significant difference in the marginal prevalence of post-SDT ≥ 4 between the groups. In patients with pre-SDT ≥ 4, the marginal prevalence (78.6–42.4% = 36.2%) was significantly different from those with pre-SDT < 4 (22.7–0.0% = 22.7%).

**Table 4:** The prevalence of post-DT ≥ 4 between music group and non-music group, stratified by the level of pre-SDT

Cochran Q = 9.87, *p* = 0.0072	Pre-DT ≥ 4			Pre-DT < 4		
	Music group	Non-music group	p value^‡^	Music group	Non-music group	p value^‡^
N	33	28		17	22	
n(% ) of post-SDT ≥ 4	14(42.4%)	22(78.6%)	0.0042	0(0.0%)	5(22.7%)	0.0565

^‡^ The p value indicated the significance of heterogeneity of high post-DT prevalence between music group and no-music group by chi-square test and stratified by the level of pre-DT. The Cochran-Mantel-Haenszel Statistics was performed, the Cochran Q was 9.87 and *p* = 0.0072, the result indicated the marginal prevalence (78.6%-42.4%=36.2%) of post-DT ≥ 4 between groups in patients had pre DT ≥ 4 was significantly different from (22.7%-0.0%=22.7%) in the patients had pre DT < 4

### Outcome of the brief symptom rating scale

**Fig. 3:**
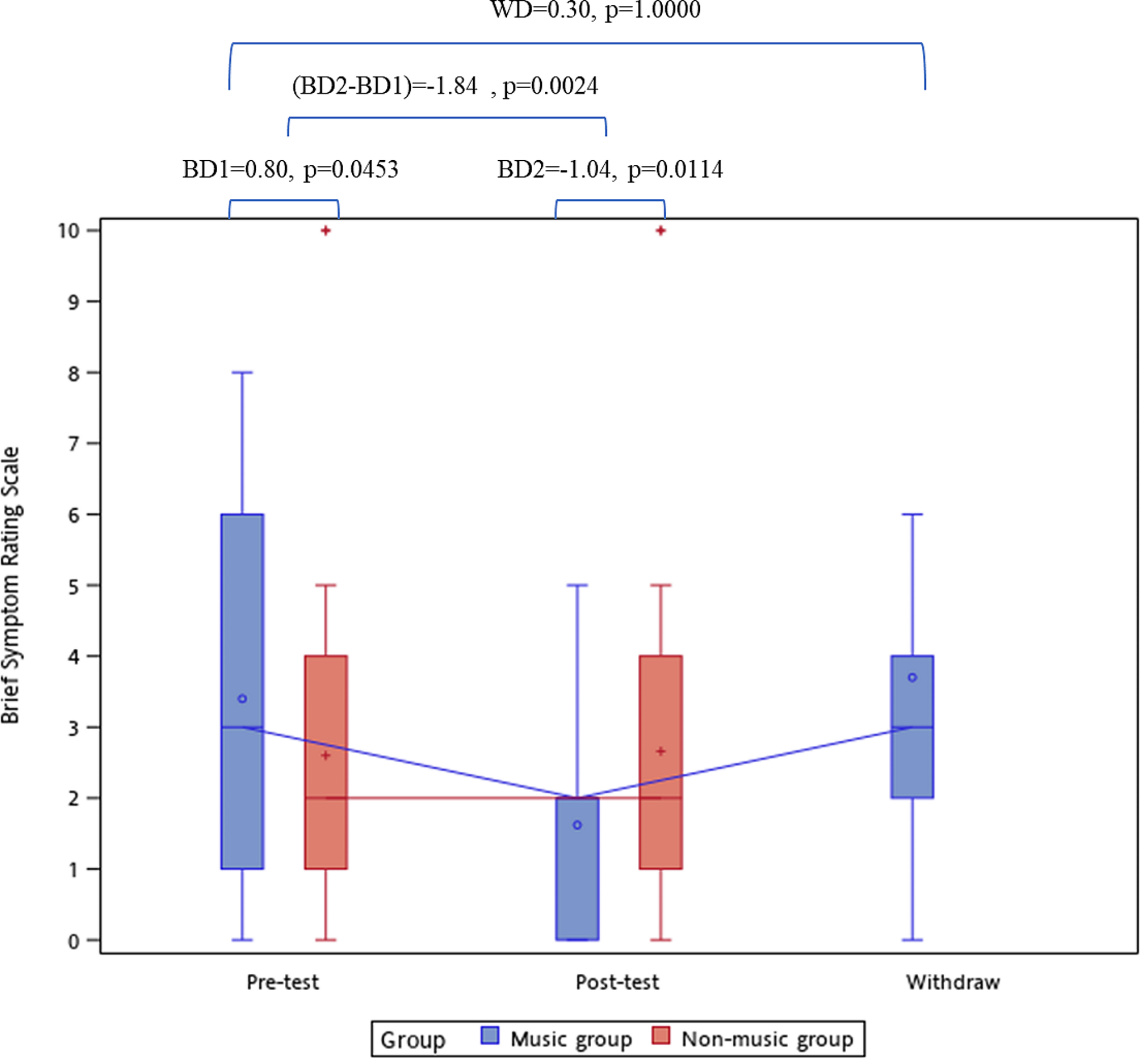
The between-group and within-group difference of BSRS-5. BD1, the difference of BSRS-5 between music group and non-music group during pre-test, and compared by Mann-Whitney U test. BD2, the difference of BSRS-5 between music group and non-music group during post-test, and compared by Mann-Whitney U test. (BD2-BD1), the pre-post difference of BSRS-5 between music group and non-music group, and estimated by generalized linear mixed model. WD, the difference of BSRS-5 in music group after withdraw the background music listening compared with pre-test, and estimated by Wilcoxon’s signed rank test

The pre-test BSRS-5 scores were 3.4 ± 2.3 in the music group and 2.6 ± 2.3 in the control group, yielding a group difference of + 0.80 (*p* = 0.0815). The posttest BSRS-5 scores were 1.6 ± 1.4 in the intervention group and 2.7 ± 2.2 in the control group, resulting in a group difference of − 1.04 (*p* = 0.0057, Table 2). Figure 3 demonstrates that the pre–post BSRS-5 difference was − 1.84 (*p* = 0.0024) between the groups. When the music listening intervention was withdrawn, the BSRS-5 rebounded to 3.7 ± 3.0 in the music group, which was not significantly different from the pre-test BSRS-5 score (*p* = 0.9300).

### Outcome of physiological symptoms assessment

**Fig. 4:**
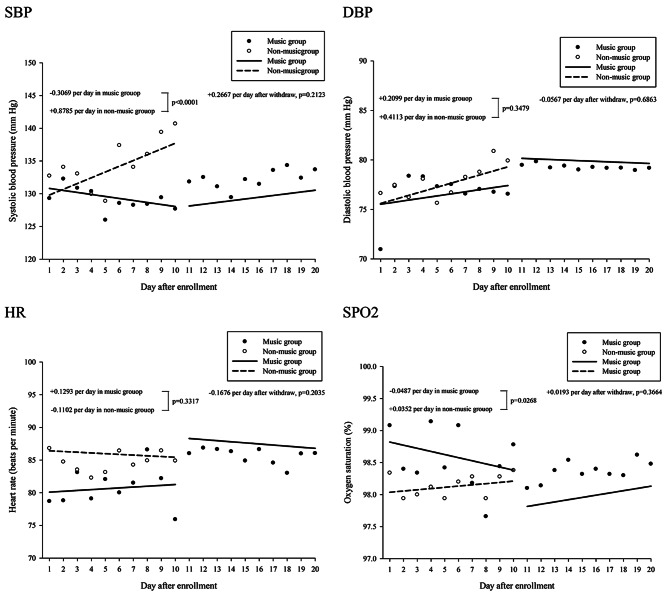
The daily change of SBP, DBP, HR, and SPO2 after radiotherapy. The difference between music group and non-music group within 10 days after first, and the daily change between 11–20 days were estimated when withdraw the background music listening

Figure 4 illustrates the daily changes in the systolic blood pressure (SBP), diastolic blood pressure (DBP), heart rate (HR), and peripheral oxygen saturation (SPO_2_) after the starting RT. During the first 10 days, the SBP in the music group decreased by − 0.3069 daily but increased by + 0.8785 in the control group. The daily change in the SBP was significantly different between the two groups (*p* < 0.0001). Once the music listening intervention was withdrawn, the daily change in the SBP in the music group was + 0.2667 (*p* = 0.2123). The daily DBP increased by + 0.2099 in the music group and + 0.4113 in the control group, with no significant difference between the two groups (*p* = 0.3479). After the music listening intervention was withdrawn, the daily change in the DBP in the music group was − 0.0567 (*p* = 0.6863). The daily HR change was + 0.1293 in the music group and − 0.1102 in the control group, with no significant difference between the two groups (*p* = 0.3317). After the music listening intervention was withdrawn, the daily HR change in the music group was − 0.1676 (*p* = 0.2035). Finally, the daily change in SPO_2_ was − 0.0497 in the music group and + 0.0352 in the control group, with a significant difference observed between the two groups (*p* = 0.0268). After the music listening intervention was withdrawn, the daily change in SPO_2_ in the music group was + 0.0193 (*p* = 0.3664).

### Study limitations

This study, while valuable, has several limitations worth considering. First, given the time constraints and demands of clinical practice, the evaluation of the music listening intervention was incomplete. We could not fully assess the effectiveness of the music listening over the entire RT process, potentially missing important insights and outcomes. Second, the application of the study was restricted, and the music options provided were limited, which could impact the generalizability of our findings. The study’s narrow scope and inability to provide a diverse range of music options limit its applicability to a broader range of patients and settings. This limitation should be considered when interpreting the study results. Third, considering that the analysis was primarily focused on a 10-day period, we failed to capture the potential long-term effects of the music intervention. As RT treatments typically extend over several weeks, our results may not reflect the sustained benefits of the intervention or changes in patient well-being over time. In addition, the potential influence of treatment-related toxicities and side effects on mental health is possible but not included in this study and needs to be investigated in the future. Finally, potential variations in the effects of music listening intervention throughout the treatment process were not fully captured. Given that our analysis was restricted to a specific period, potential fluctuations in the patients’ emotional responses and treatment experiences throughout their entire RT journey could not be evaluated. Future studies should address these limitations and implement relevant improvements. A more comprehensive understanding of the effects of music listening intervention in patients receiving RT can be achieved by examining both short- and long-term outcomes, variations in patient responses, and effects of a wider range of music options. This will make our research findings more representative of and beneficial to a broader range of patients undergoing RT.

## Discussion

The primary aim of this study is to assess the impact of background music listening on anxiety reduction among patients undergoing their initial radiotherapy. However, the enrolled patients could be categorized into three groups: those who received neoadjuvant chemotherapy, concurrent chemoradiotherapy, or radiotherapy alone. Exploring the potential differential effects of music listening based on treatment type represents a valuable direction for future research. Regarding the short-term effects of music on anxiety and stress, the existing literature reveals a trend toward a reduction in the anxiety and stress levels. Both the type of music used and timing of the intervention play crucial roles in determining the effects of music, particularly in relation to the subjective factors and musical preferences of the participants [37]. Moreover, questionnaire data underscore the subjective impact of algorithmic music as reported by the participants [38]. The incorporation of music intervention during treatment sessions has been demonstrated to have a positive influence on postchemotherapy anxiety. Specifically, patients who initially scored high on state anxiety experienced a more significant reduction in postchemotherapy anxiety when receiving music intervention, compared to subjects with normal initial state anxiety scores [37]. Our results confirm our primary hypothesis that instrumental music significantly reduces anxiety over 10 consecutive days of RT treatment. Our findings align with the results of Rossetti et al., who showed that music introduced before treatment significantly reduced patient anxiety and distress during the simulation procedure. However, two interventions were employed in their music group [11]. Conversely, our findings differ from those of O’Steen et al., who concluded that music delivered via a web-based platform did not meaningfully reduce anxiety during the first radiation treatment [11].

Distress is an important vital sign in cancer patients [26], and historically, strategies such as talking, communication, or group therapy have been employed to manage it. A systematic review of distress management in cancer patients found that 6 out of 10 trials analyzed showed significant improvements in distress [39]. This study’s significantly reduced prevalence of post-SDT ≥ 4 in the music listening intervention group (Table 4) indicates significant improvements in distress. These findings suggest that music listening interventions could serve as an effective non-pharmacological treatment to improve distress in cancer patients.

Given the clinical practice limitations in our hospital, involving patients in music selection is challenging. Consequently, music for patients’ listening without their input [40, 41]. Harper et al. explored the impact of self-selected music listening during chemotherapy infusion, demonstrating its positive effects on reducing negative mood and distress scores [42]. Investigating the effects of self-selected music listening in RT would be worthwhile in future studies. In our study, the enrolled cancer patients also exhibited a significant number of underlying mood disorders, which is similar to Chernecky’s study that reported significantly high levels of anxiety and distress among cancer patients undergoing treatment. This may be due to uncertainty regarding the treatment outcomes and lack of prior experience with RT [43]. To thoroughly investigate the potential of music listening as an intervention for reducing physiological parameters such as BP and HR, and alleviating anxiety, it is imperative to conduct larger randomized controlled trials. These studies should include a substantial number of participants and focus on specific healthcare settings to ensure the relevance and applicability of the findings [44].

## Conclusions

In conclusion, our study demonstrates that background music listening effectively reduces anxiety symptoms among cancer patients undergoing RT. Our findings contribute to the mounting evidence advocating for the utilization of music listening intervention as a noninvasive, safe, and cost-effective approach for enhancing patient well-being. The evidence suggests that music interventions can play a pivotal part in improving the healthcare of cancer patients. Such interventions serve as an innovative and comforting strategy for mitigating the emotional and psychological burdens associated with intense cancer treatments, such as RT. We hope that our findings promote improvements in the quality of care provided to cancer patients. It is our earnest aspiration to inspire healthcare providers to consider incorporating music interventions into their clinical practice. By embracing such therapeutic options, we can offer a more holistic and patient-centered approach to the often daunting journey of cancer treatment. Further research is recommended to explore the long-term effects of music interventions and establish standardized protocols for their implementation in clinical settings. The results of this study, and those that may follow, serve to underscore the profound potential of harnessing the power of music listening within a therapeutic context.

## Data Availability

No datasets were generated or analysed during the current study.

## Abbreviations

BAI: Beck Anxiety Inventory
DBP: Diastolic blood pressure
DT: Distress Thermometer
HR: Heart rate
RT: Radiation therapy
SBP: Systolic blood pressure
SDT: Symptom Distress Thermometer
